# Relationship between Arterial Stiffness and Renal Function Determined by Chronic Kidney Disease Epidemiology Collaboration (CKD-EPI) and Modification of Diet in Renal Disease (MDRD) Equations in a Chinese Cohort Undergoing Health Examination

**DOI:** 10.1155/2022/8218053

**Published:** 2022-03-14

**Authors:** Biwen Tang, Weichao Tu, Jiehui Zhao, Xueqing Deng, Isabella Tan, Mark Butlin, Alberto Avolio, Junli Zuo

**Affiliations:** ^1^Department of Geriatrics, Ruijin Hospital, Shanghai Jiao Tong University School of Medicine, China; ^2^Department of Urology, Ruijin Hospital, Shanghai Jiao Tong University School of Medicine, China; ^3^Daning Community Health Service Center, Shanghai, China; ^4^Macquarie Medical School, Faculty of Medicine, Health and Human Sciences, Macquarie University, Sydney, Australia; ^5^The George Institute for Global Health, Sydney, Australia

## Abstract

**Background:**

The association between arterial stiffness and cardiovascular risk in CKD and ESRD patients is well established. However, the relationship between renal function estimation and properties of large arteries is unclear due to the four different methods used to quantify glomerular filtration. This study investigated the relationship between carotid-femoral pulse wave velocity (c-fPWV), as a measure of arterial stiffness, and accepted metrics of renal function.

**Methods:**

This cross-sectional study was conducted in 431 health examination individuals in China, enrolled from January 2017 to June 2019. c-fPWV and blood pressure were measured, and blood samples were obtained for all participants. Four different methods were used to determine the estimated glomerular filtration rate (eGFR) as described by the Chronic Kidney Disease Epidemiology Collaboration (CKD-EPI) and Modification of Diet in Renal Disease (MDRD) equations: (i) CKD-EPI_SCr_ formula based on SCr, (ii) CKD-EPI_CysC_ formula based on CysC, (iii) CKD-EPI_SCr/CysC_ formula based on Cr and CysC, and (iv) MDRD.

**Results:**

Of all of the study participants (average age 53.1 ± 13.0 years, 68.1% male), 23.7% had diabetes mellitus and 66.6% had hypertension. The average eGFR values determined by the CKD-EPI_SCr_, CKD-EPI_CysC_, CKD-EPI_SCr/CysC_, and MDRD equations were 91.9 ± 15.6, 86.8 ± 21.4, 89.6 ± 18.3, and 90.7 ± 16.6 ml/min/1.73m^2,^ respectively. c-fPWV was significantly and negatively correlated with eGFR determined by CKD-EPI_SCr_ (*r* = −0.336, *P* < 0.001), CKD-EPI_CysC_ (*r* = −0.385, *P* < 0.001), CKD-EPI_SCr/CysC_ (*r* = −0.378, *P* < 0.001), and MDRD (*r* = −0.219, *P* < .001) equations. After adjusting for confounding factors, c-fPWV remained significantly and negatively correlated with eGFR determined by the CKD-EPI_CysC_ equation (*β* = −0.105, *P* = 0.042) and significantly and positively correlated with age (*β* = 0.349, *P* ≤ 0.01), systolic pressure (*β* = 0.276, *P* ≤ 0.01), and hypoglycemic drugs (*β* = 0.101, *P* = 0.019).

**Conclusion:**

In a health examination population in China, c-fPWV is negatively correlated with eGFR determined by four different equations; however, only the metric of eGFR determined by the equation for CKD-EPI_CysC_ showed an independent relation with c-fPWV.

## 1. Introduction

The prevalence of chronic kidney disease (CKD) is increasing every year and has become a global public health problem. The prevalence in the general population worldwide has reached 14.3% [1]. Data from China suggests that the prevalence of CKD among people over 18 years old is 10.8% [2]. Based on China's large total population, there are more than 100 million CKD patients. CKD patients face a high risk of cardiovascular disease. A Chinese study [3] showed that 27.8% of Chinese hospitalized CKD patients had cardiovascular disease, of which coronary heart disease was the most common (17.7%), followed by heart failure (13.0%) and stroke (9.2%). The risk of cardiovascular death in patients with end-stage renal disease is as high as 3.0/100 (person-year) [4].

The association between arterial stiffness and cardiovascular risk in CKD patients is well established [5, 6]. The development of arterial stiffness may be related to traditional and nontraditional cardiovascular risk factors. The former includes the cumulative effects of diabetes, hypertension, smoking, and obesity. The latter includes oxidative stress, inflammation, uremic environment, such as accumulation of uremic toxins, and vascular calcification [7]. Accumulation of advanced glycosylation end products, increased collagen cross-linking, and activation of the renin–angiotensin system and other mechanisms can also cause arterial stiffness to increase in patients with impaired renal function [8]. In the early stages of CKD, the structure and mechanical properties of the aorta change, and there is enlargement of the common carotid artery and increased aortic stiffness [9].

There are many formulas for calculating renal function in CKD patients, and the correlation between different formulas and arterial stiffness is still controversial. A study has suggested that the relationship between estimated glomerular filtration rate (eGFR) and arterial stiffness is nonlinear [10]. Moderate to severe renal impairment is associated with increased arterial stiffness. But the correlation between mild renal impairment and arteriosclerosis is more uncertain [11, 12]. We aim to clarify the sensitivity formula for calculating renal function in a health examination population.

Carotid-femoral pulse wave velocity (c-fPWV) is an effective noninvasive measure of arterial stiffness and can predict adverse cardiovascular outcomes and all-cause mortality in unselected populations [13–15]. Our study investigated the relationship between c-fPWV and renal function in a health examination population and observed the correlation between early renal impairment and c-fPWV. Because eGFR's test results are affected by many factors, our study adopted four different formulas for evaluation and sought the most representative test method.

## 2. Methods

### 2.1. Study Population

This is a cross-sectional study conducted in a health examination population at Ruijin Hospital from January 2017 to June 2019. c-fPWV was measured, and blood samples were taken in all participants. Inclusion criteria are as follows: (1) age ≥18 years old; (2) agree to participate in this study and sign an informed consent form. Exclusion criteria are as follows: (1) patients with valvular disease and cardiomyopathy; (2) patients with atrial fibrillation, atrioventricular, and intraventricular block; (3) cardiovascular or cerebrovascular disease in the past 3 months; and (4) myocardial infarction, chronic heart failure in the past 3 months; and (5) tumor patients.

### 2.2. Measurement of c-fPWV

c-fPWV was measured using applanation tonometry with a Millar transducer and SphygmoCor software (AtCor Medical, Sydney, Australia). The c-fPWV measurement was performed by placing the transducer at the femoral and then the carotid artery. Distance was measured on the body surface from the suprasternal notch to femoral and carotid artery sites, and the subtraction distance method was used to determine cfPWV from the foot-to-foot pulse transit time between the carotid and femoral pulses in reference to the R wave of the electrocardiogram.

### 2.3. Determination of Cystatin C and Serum Creatinine

Cystatin C was measured by colloidal gold colorimetric method. Creatinine was measured using the picric acid method. Both were determined by Beckman Coulter AU5800 automatic biochemistry analyzer.

### 2.4. Evaluation of eGFR

In 2012, the Kidney Diseases Improving Global Outcomes (KDIGO) guidelines recommend the use of Chronic Kidney Diseases Epidemiology Collaboration (CKD-EPI) formula for eGFR to assess renal function. This series of formula is based on creatinine (Cr) and cystatin C (CysC), mainly including CKD-EPI_SCr_ formula based on Cr, CKD-EPI_CysC_ formula based on CysC, and CKD-EPI_SCrCysC_ formula based on Cr and CysC [16]. Each equation for eGFR is shown below [17, 18] (GFR[mL/min/1.73m^2^]; SCr(umol/L); CysC: (mg/L)):

- MDRD

GFR = 186 × (SCr)^−1.154^ × age^−0.203^ × 0.742 (female)

(b) CKD-EPI_SCr_
  1. GFR = 144 × (SCr/62) − 0.329 × 0.993^age^(SCr ≤ 62, female)
  2. GFR = 144 × (SCr/62) − 1.209 × 0.993^age^(SCr > 62, female)
  3. GFR = 141 × (SCr/80) − 0.411 × 0.993^age^(SCr ≤ 80, male)
  4. GFR = 141 × (SCr/80) − 1.209 × 0.993^age^(SCr > 80, male)
(c) CKD-EPI_CysC_
  1. GFR = 133 × (CysC/0.8) − 0.499 × 0.996^age^ × 0.932(CysC ≤ 0.8, female)
  2. GFR = 133 × (CysC/0.8) − 1.328 × 0.996^age^ × 0.932(CysC > 0.8, female)
  3. GFR = 133 × (CysC/0.8) − 0.499 × 0.996^age^(CysC ≤ 0.8, male)
  4. GFR = 133 × (CysC/0.8) − 1.328 × 0.996^age^(CysC > 0.8, male)
(d) CKD-EPI_SCr-cysC_
  1. GFR = 130 × (SCr/62) − 0.248 × (CysC/0.8) − 0.375 × 0.995^age^(SCr ≤ 62, CysC ≤ 0.8, female)
  2. GFR = 130 × (SCr/62) − 0.248 × (CysC/0.8) − 0.711 × 0.995^age^(SCr ≤ 62, CysC > 0.8, female)
  3. GFR = 130 × (SCr/62) − 0.601 × (CysC/0.8) − 0.375 × 0.995^age^(SCr > 62, CysC ≤ 0.8, female)
  4. GFR = 130 × (SCr/62) − 0.601 × (CysC/0.8) − 0.711 × 0.995^age^(SCr > 62, CysC > 0.8, female)
  5. GFR = 135 × (SCr/80) − 2.07 × (CysC/0.8) − 0.375 × 0.995^age^(SCr ≤ 80, CysC ≤ 0.8, male)
  6. GFR = 135 × (SCr/80) − 2.07 × (CysC/0.8) − 0.711 × 0.995^age^(SCr ≤ 80, CysC > 0.8, male)
  7. GFR = 135 × (SCr/80) − 0.601 × (CysC/0.8) − 0.375 × 0.995^age^(SCr > 80, CysC ≤ 0.8, male)
  8. GFR = 135 × (SCr/80) − 0.601 × (CysC/0.8) − 0.711 × 0.995^age^(SCr > 80, CysC > 0.8, male)

### 2.5. Definition of Hypertension, Diabetes Mellitus, and Dyslipidemia

Hypertension is defined as office SB *P* values at least 140 mmHg and/or diastolic BP (DBP) values at least 90 mmHg [19], or currently known use of antihypertensive medication. The criteria for the diagnosis of diabetes are as follows: fasting plasma glucose (FPG) ≥ 126 mg/dL (7.0 mmol/L). Fasting is defined as no caloric intake for at least 8 h or 2 h plasma glucose (PG) ≥ 200 mg/dL (11.1 mmol/L) [20], or currently known use of hypoglycemic agents. Total cholesterol (TC) ≥ 5.2 mmol/L or hypertriglyceridemia (TG) ≥ 1.7 mmol/L diagnosis dyslipidemia [21], or currently known lipid-lowing therapy. We defined no drinking as never consuming alcohol.

### 2.6. Statistical Analysis

Continuous variables are presented as mean ± SD. Pearson test was used to evaluate the correlation between normally distributed univariate variables and c-fPWV. A two-sided *P* < 0.05 was considered statistically significant throughout the analyses. The association of eGFR with c-fPWV was assessed by means of linear regression. Linear regression statistics were used to compare the slope of the lines by using standardized coefficients of eGFR in four formulas and c-fPWV. The analyses were performed using SPSS, version 17.0 (SPSS, Chicago, IL). The nonlinear regression correlation analysis between eGFR and c-fPWV was also performed by using univariate curve fitting analyses and the performance of the simple linear regression model, and the quadratic regression model for CKD-EPI_CysC_ was compared by ANOVA test using R software (4.1.2).

### 2.7. Ethics Statement

All studies were in compliance with the Declaration of Helsinki, Good Clinical Practice guidelines, and applicable regulatory requirements. All participants provided written informed consent to participate for the respective study, which was approved by the Human Research Ethics Committee at Ruijin Hospital, Shanghai Jiao Tong University School of Medicine.

## 3. Results

We enrolled 431 participants in our study. The characteristics of participants are shown in Table 1. The average age of the 431 participants was 53.1 ± 13.0 years, and 68.1% were male. The average BMI was 25.8 ± 4.1 kg/m^2^. A total of 23.7% of the study participants had diabetes mellitus. 287 (66.6%) participants had hypertension. 32.3% (139) participants were smokers. The average values of eGFR for CKD-EPI_SCr_, CKD-EPI_CysC_, CKD-EPI_SCr/CysC_, and MDRD equations were 91.9 ± 15.6, 86.8 ± 21.4, 89.6 ± 18.3, and 90.7 ± 16.6 (ml/min/1.73m^2^), respectively.

The participants were divided into two groups based on eGFR. The c-fPWV values were significantly higher in the group with moderately reduced eGFR (eGFR ≤ 60 ml/min/1.73m^2^) than the mildly reduced group for the different equations (Table 2).

**Figure 1:**
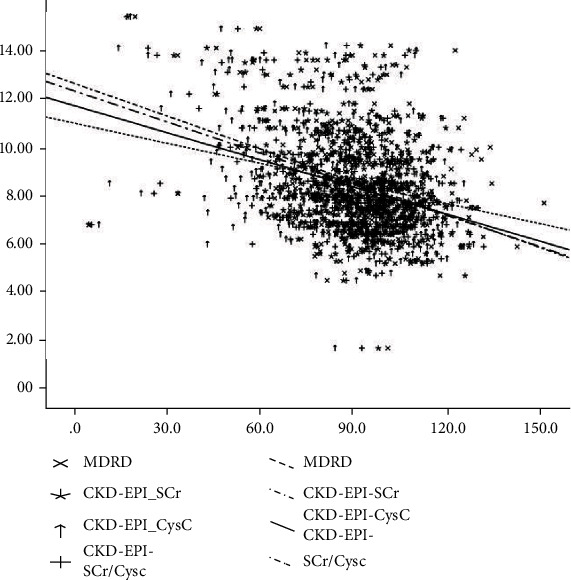
The correlation between estimated glomerular filtration rate (eGFR) and carotid-femoral pulse wave velocity (c-fPWV) for different eGFR equations. See text for abbreviation definitions.

Pearson correlation showed that c-fPWV was significantly and negatively correlated with eGFR of CKD-EPI_SCr_ (*r* = −0.336, *P* < 0.001), CKD-EPI_CysC_ (*r* = −0.385, *P* < 0.001), CDK-EPI_SCr/CysC_ (*r* = −0.378, *P* < 0.001), and MDRD (*r* = −0.219, *P* < 0.001) equations. In addition, study groups based on age showed that c-fPWV was significantly and negatively correlated with eGFR of CKD-EPI_SCr_ (*r* = −0.330, *P* < .001), CKD-EPI_CysC_ (*r* = −0.338, *P* < 0.001), CDK-EPI_SCr/CysC_ (*r* = −0.349, *P* < 0.001), and MDRD (*r* = −0.265, *P* < 0.001) equations in age ≥60 years (Table 3).  Figure 1 shows that the equation of CKD-EPI_CysC_ has higher correlation than the others (*r* = −0.385, *P* < 0.001). We further performed the univariate curve fitting analyses to evaluate the correlations between CKD-EPI_CysC_, CKD-EPI_SCr/CysC_, CKD-EPI_SCr_, MDRD, and c-fPWV in health examination individuals. We noticed that the adjusted R^2^ values for the regression models were significantly reduced in the nonlinear models (including logarithmic model, exponential model, power model, inverse model, exponential model, and cubic regression model) except for the quadratic regression models. Furthermore, we found the coefficients for the quadratic terms were not significant except MDRD (see Table 3). We also performed the ANOVA test to compare the performance of the simple linear regression model and quadratic regression model for CKD-EPI_CysC_ that showed strongest correlation with c-fPWV, and no significance difference for the regression performance was seen (*P* = 0.1932) (Table. S1 and Figure S1 in Supplementary Section).

Stepwise multivariate regression analysis (model 1, adjusted for age, SBP, HR, eGFR (four equations), LDL, and FPG) showed the predictors of c-fPWV, with c-fPWV considered as the dependent variable. c-fPWV was significantly and negatively correlated with eGFR (*β* = −0.110, *P* = 0.027) evaluated by CKD-EPI_CysC_ equation. Otherwise, c-fPWV was significantly and positively correlated with age (*β* = 0.378, *P* < .001), SBP (*β* = 0.278, *P* < .001), HR (*β* = 0.094, *P* = 0.023), and FPG (*β* = 0.105, *P* = 0.019). Further model (model 2, adjusted for age, SBP, HR, eGFR (four equations), LDL, FPG, and smoking) c-fPWV was also significantly and negatively correlated with eGFR (*β* = −0.118, *P* = 0.018) evaluated by CKD-EPI_CysC_ equation. c-fPWV was also significantly and negatively correlated with eGFR (*β* = −0.01, *P* = 0.042) evaluated by CKD-EPI_CysC_ equation in model 3 (adjusted for age, SBP, HR, eGFR, LDL, FPG, smoking, antihypertensive medication, statins, and hypoglycemic agents) (Table 4).

## 4. Discussion

Glomerular filtration rate (GFR) is an independent predictor of kidney injury, all-cause death, cardiovascular death, and renal failure [22]. Given the close correlation between CKD and cardiovascular disease (CVD), early detection of renal dysfunction is important to improve the risk stratification of atherosclerotic disease.

In this study, we investigated the relationship between arterial stiffness as measured by c-fPWV and renal function in a population undergoing health assessment. Irrespective of the eGFR formula used, c-fPWV is significantly and negatively correlated with eGFR. This result is consistent with previous studies [23, 24].

A large sample study also found that arterial stiffness is related with the decline in renal function, and vascular stiffness could be a target for delaying decline in eGFR. Each SD of higher c-fPWV was associated with 7% greater risk of incident CKD [25]. Adequate BP and c-fPWV control can affect long-term BP reduction, and more cardiovascular survival is observed [26]. Mourad et al. found that increased stiffness of central arteries was statistically associated with reduced creatinine clearance in subjects with mild-to-moderate renal disease [27].

The relationship between renal function and arterial stiffness may be bidirectional. Phosphate retention in CKD patients and calcification of human aortic smooth muscle cells leads to increase in large artery stiffness [26]. Several factors such as oxidative stress, inflammation, and anemia in CKD patients might influence arterial structure and lead to arterial remodeling and stiffening [28, 29]. In addition, the renal vasculature has the characteristics of low resistance and impedance. Once blood pressure rises or arterial stiffness increases, the kidney will passively receive high perfusion and high pulsatile blood flow, leading to potential damage of the renal arteries and capillaries [24].

In our study, all renal function formulas showed a significant correlation. It should be noted that some studies have not found a link between mild renal impairment and arterial stiffness [10, 11]. These two studies, respectively, adopted the Japanese Society of Nephrology model and MDRD formula to calculate eGFR. Different renal function equations have different sensitivity and specificity in different people. This may be the reason for the inconsistent results. The current guidelines recommend using the CKD-EPI equation to assess the renal function of adult CKD patients [16]. CKD-EPI has a smaller standard deviation than MDRD and has a higher precision and accuracy in people with GFR ≥ 60 mL/min/1.73 m^216^. The MDRD formula has the highest accuracy in patients with moderate to severe renal impairment, but it decreases with improved renal function [9], as in our population. Although the use of exogenous substances (for example, 99mTechnetium-diethylenetriamine pentaacetic acid, 99mTc-DTPA) to determine GFR is the most accurate, it is difficult to carry it out routinely in clinical practice due to cost and resources [30]. Serum creatinine and cysteine protease inhibitor C are both endogenous molecules. Serum creatinine is unstable and easily influenced by daily diet, secretion and reabsorption of renal tubular cells, and reduced muscle mass which is common in CKD patients [31].

Cystatin C is considered to be an ideal endogenous GFR marker that is more sensitive than serum creatinine and can reflect the filtration function of the kidney [32]. Cystatin C is not affected by factors, such as gender, age, diet, inflammation, and muscle mass, and is often significantly abnormal early in the course of the disease.

A study in a Chinese population suggested that the eGFR equation combined with cystatin C is superior to eGFR based on creatinine in early detection of kidney injury, and CKD-EPI_CysC_ is more sensitive to detect kidney injury and predict kidney outcome [31]. Another study of men in the community suggested that CKD-EPI_CysC_ is the formula of choice to predict death in community-dwelling older men [33]. Based on the cost-effectiveness and accessibility of cystatin tests, the CKD-EPI_CysC_ formula is more accurate to estimate GFR in a population with normal to mildly reduced renal function; the correlation between arterial stiffness and true GFR still requires further investigation. It should be noted that the CKD-EPI_CysC_ method is more suitable for the detection of renal function in nondialysis patients, but not for dialysis patients [34].

Our study also found that c-fPWV was significantly higher in people with moderately reduced GFR than those with mildly decline ones. Briet et al. [35] study also showed that c-fPWV was significantly higher in CKD stage 2-5 patients than in hypertensives and normotensives. Arterial enlargement and increased arterial stiffness occur in patients with mild-to-moderate CKD. It is speculated that with the obvious decline of renal function, the effect on the structure and function of arteries will be longer and the damage will be more obvious.

This study has some limitations. Firstly, it was a small cross-study study, and so, it is difficult to explicitly distinguish associations and causality. The results need to be further confirmed in large prospective studies. Secondly, the study examines an Asian population, and so, findings may not necessarily be extrapolated to other ethnic groups. Third, the results of serum of creatine in all equations, even the CKD-EPI equations, was measured with the picric acid method that would be higher than the enzymatic method; therefore, it would overestimate the eGFR. Fourth, diabetic patients are not excluded; otherwise, the sample size would be reduced in this study, but it will be considered in future studies. Finally, as subjects were undergoing health assessment, findings will need to be further explored in future studies using different formulas for renal function and measuring arterial stiffness in patients with CKD.

## 5. Conclusions

In a health examination population in China, c-fPWV is negatively correlated with eGFR evaluated in different four equations. The equation of CKD-EPI_CysC_ had higher correlation than others and also showed an independent relation with c-fPWV.

## Figures and Tables

**Table 1:** Demographic characteristics of the population (*n* = 431).

Parameter	Mean ± SD
Age (years)	53.1 ± 13.0
BMI (kg/m^2^)	25.8 ± 4.1
WHR	0.9 ± 0.1
Sex	
Male (%)	294 (68.1%)
Female (%)	137 (31.9%)
SBP (mmHg)	131 ± 18
DBP (mmHg)	76 ± 12
HR (bpm)	67 ± 10
IMT (mm)	0.7 ± 0.2
Cystatin C (mg/L)	1.0 ± 0.4
LDL-c (mmol/L)	3.2 ± 1.6
FPG (mmol/L)	5.9 ± 2.0
Scr (umol/L)	
Male	84.0 ± 19.5
Female	65.5 ± 8.4
eGFR (mL/min/1.73m^2^)	
CKD-EPI_SCr_	91.9 ± 15.6
CKD-EPI_CysC_	86.8 ± 21.4
CKD-EPI_SCr/CysC_	89.6 ± 18.3
MDRD	90.7 ± 16.6
LVM (g)	190.5 ± 57.9
LVMI (g/m)	104.0 ± 26.8
c-fPWV (m/s)	8.4 ± 2.1
DM (%)	102 (23.7%)
HTN (%)	287 (66.6%)
Dyslipidemia (%)	194 (45.0%)
Smoking (%)	139 (32.3%)
Drink (%)	123 (28.5%)
Statin (%)	170 (39.4%)
Aspirin (%)	48 (11.1%)
Antihypertensive medication	
ACEI/ARB	198 (69.0%)
CCB	119 (41.5%)
Diuretic	14 (4.9%)
*β*-Blocker	37 (12.9%)

BMI: body mass index; WHR: waist-hip ratio; SBP: systolic blood pressure; DBP: diastolic blood pressure; HR: heart rate; Scr: serum creatinine; IMT: intima-media thickness; eGFR: estimated glomerular filtration rate; LVM: left ventricular mass; LVMI: left ventricular mass index; c-fPWV: carotid-femoral pulse wave velocity; DM: diabetes mellitus; HTN: hypertension.

**Table 2:** Relationship between GFR and c-f PWV.

	c-fPWV (eGFR ≥ 60)	c-fPWV (eGFR<60)	*P* value
CKD-EPI_SCr_	8.3 ± 2.0	12.1 ± 2.6	<0.01
CKD-EPI_CysC_	8.1 ± 1.8	10.4 ± 2.8	<0.01
CKD-EPI_SCr/CysC_	8.2 ± 2.0	10.7 ± 2.7	<0.01
MDRD	8.3 ± 2.0	12.0 ± 2.8	<0.01

**Table 3:** Pearson correlation among variables.

	c-fPWV	CKD-EPI_SCr_	CKD-EPI_CysC_	CKD-EPI_SCr/CysC_	MDRD
c-fPWV					
CKD-EPI_SCr_	-0.336^∗∗^				
CKD-EPI_CysC_	-0.385^∗∗^	0.763^∗∗^			
CKD-EPI_SCr/CysC_	-0.378^∗∗^	0.898^∗∗^	0.996^∗∗^		
MDRD	-0.219^∗∗^	0.917^∗∗^	0.696^∗∗^	0.830^∗∗^	
c-fPWV					
Age ≥ 60		-0.330^∗∗^	-0.338^∗∗^	-0.349^∗∗^	-0.265^∗∗^
Age < 60		-.028	-0.104	-0.053	0.043

^∗∗^ *P* < 0.01.

**Table 4:** Determinants of c-fPWV.

Variable	B	*β*	Se	*P* value	Adjusted R^2^
Moldel 1					0.328
(constant)	0.370				
Age	0.063	0.378	0.008	<0.01	
SBP	0.033	0.278	0.005	<0.01	
HR	0.020	0.094	0.009	0.023	
CKD-EPI_CysC_	-0.011	-0.110	0.005	0.027	
FPG	0.105	0.097	0.045	0.019	
Model 2					0.330
(constant)	0.671				
Age	0.06	0.347	0.008	<0.01	
SBP	0.033	0.275	0.005	<0.01	
HR	0.019	0.086	0.009	0.038	
CKD-EPI_CysC_	-0.012	-0.118	0.005	0.018	
FPG	0.107	0.098	0.045	0.019	
Model 3					0.351
(constant)					
Age	0.064	0.394	0.008	<0.01	
SBP	0.033	0.276	0.005	<0.01	
HR	0.017	0.076	0.010	0.073	
CKD-EPI_CysC_	-0.010	-0.105	0.005	0.042	
FPG	0.093	0.086	0.207	0.051	

Model 1: Adjusted for sex, age, SBP, HR, MDRD, CKD-EPI_SCr_, CKD-EPI_CysC_, CKD-EPI_SCr/CysC_, LDL, and FPG. Model 2: adjusted for sex, age, SBP, HR, MDRD, CKD-EPI_SCr_, CKD-EPI_CysC_, CKD-EPI_SCr/CysC_, LDL, FPG, and smoking. Model 3: adjusted for sex, age, SBP, HR, MDRD, CKD-EPI_SCr_, CKD-EPI_CysC_, CKD-EPI_SCr/CysC_, LDL, FPG, smoking, antihypertensive medication, statins, and hypoglycemic agents. SBP: systolic blood pressure; HR: heart rate; LDL-c: low density lipoprotein cholesterol; FPG: fasting blood glucose.

## Data Availability

The data that support the findings of this study are available from the corresponding author, [Jl Z], upon reasonable request.

## Supplementary Materials

Supplementary Materials Figure S1. the univariate curve fitting analyses to evaluate the correlations between CKD-EPI_CysC_, CKD-EPI_SCr/CysC_, CKD-EPI_SCr_, MDRD, and c-fPWV in health examination of individuals. The red lines represent the quadratic regression model; the blue lines represent the simple linear regression model. Table.S1: the ANOVA test of the simple linear regression model and quadratic regression model.
