# Supplementary material for: Relationship between Arterial Stiffness and Renal Function Determined by Chronic Kidney Disease Epidemiology Collaboration (CKD-EPI) and Modification of Diet in Renal Disease (MDRD) Equations in a Chinese Cohort Undergoing Health Examination
Source: Biomed Res Int. 2022 Mar 14;2022:8218053. doi: 10.1155/2022/8218053 (PMC8938063; doi:10.1155/2022/8218053)
Supplement: Supplementary Materials — Figure S1. the univariate curve fitting analyses to evaluate the correlations between CKD-EPICysC, CKD-EPISCr/CysC, CKD-EPISCr, MDRD, and c-fPWV in health examination of individuals. The red lines represent the quadratic regression model; the blue lines represent the simple linear regression model. Table.S1: the ANOVA test of the simple linear regression model and quadratic regression model. [file 8218053.f1.docx]

**Supplementary Section**

**Relationship between arterial stiffness and renal function determined by Chronic Kidney Disease Epidemiology Collaboration (CKD-EPI) and Modification of Diet in Renal Disease (MDRD) equations in a Chinese cohort undergoing health examination**

Biwen Tang*^, 1^, Weichao Tu^*,2^, Jiehui Zhao^3^, Xueqing Deng^1^,Tan, Isabella Tan^4^, Mark Butlin^4^, Alberto Avolio^4^ and Junli Zuo^1,4^

1. Department of Geriatrics, Ruijin Hospital, Shanghai Jiao Tong University School of Medicine.
2. Department of Urology, Ruijin Hospital, Shanghai Jiao Tong University School of Medicine.
3. Daning Community Health Service Center, Shanghai, China.
4. Macquarie Medical School, Faculty of Medicine, Health and Human Sciences, Macquarie University, Sydney, Australia.

*These authors contributed equally to this study.

**Keywords:** Pulse wave velocity, eGFR, renal function, arterial stiffness, CKD-EPI_SCr_, CKD-EPI_CysC_, CKD-EPI_SCr/CysC_, MDRD

**Running Title**: Arterial stiffness and renal function

**Address for Correspondence**:

Dr Junli Zuo, MD, PHD and Dr Jiehui Zhao

Department of Geriatrics, Ruijin Hospital, Shanghai Jiao Tong University School of Medicine.9B, No. 999, Xiwang Road, Shanghai, CHINA

Tel: +86 21 67888959

Email: zjl12616@rjh.com.cn


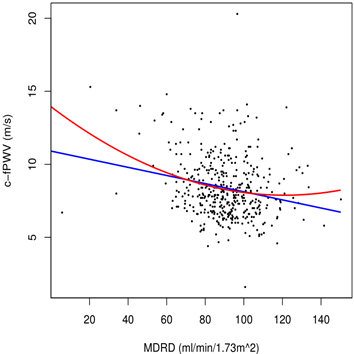

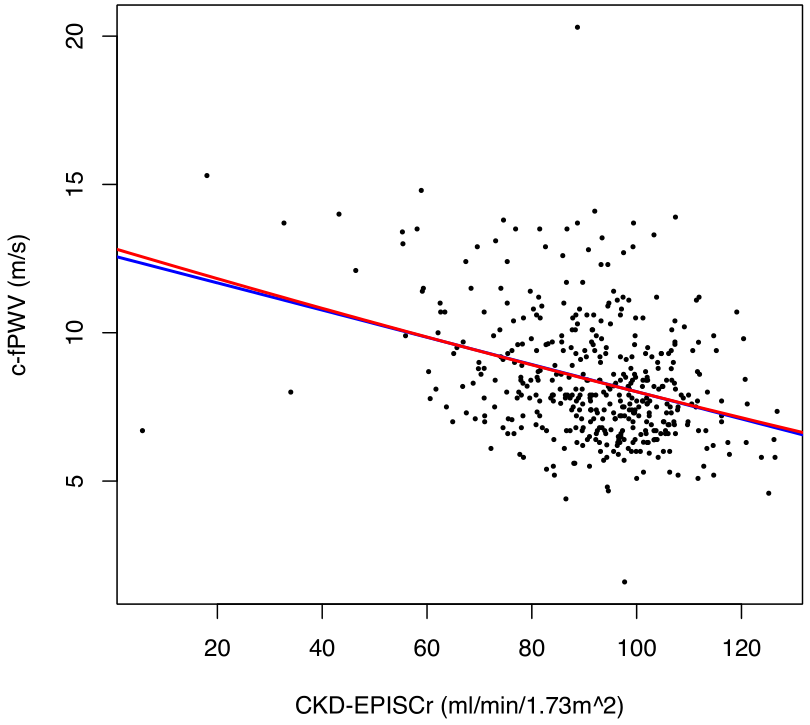


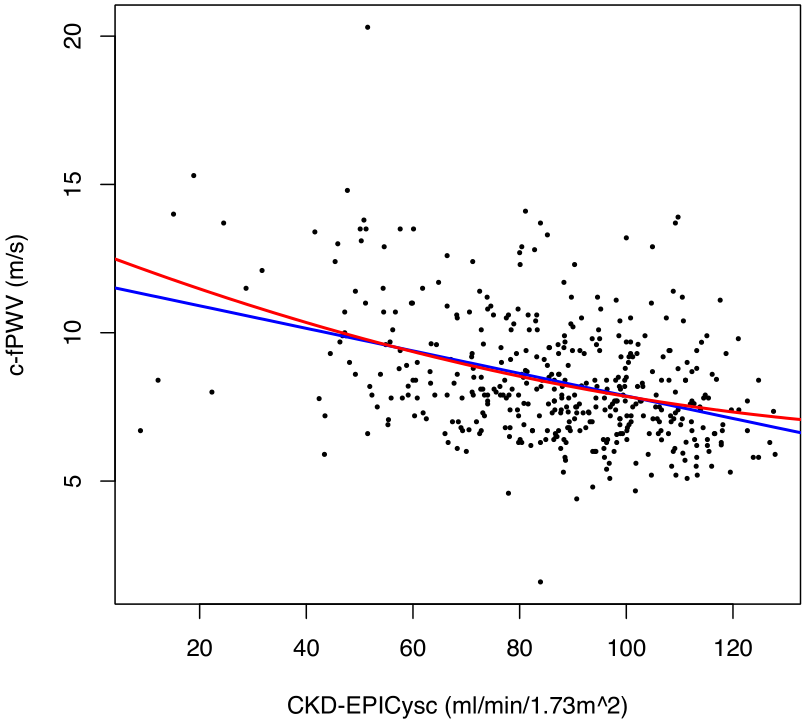

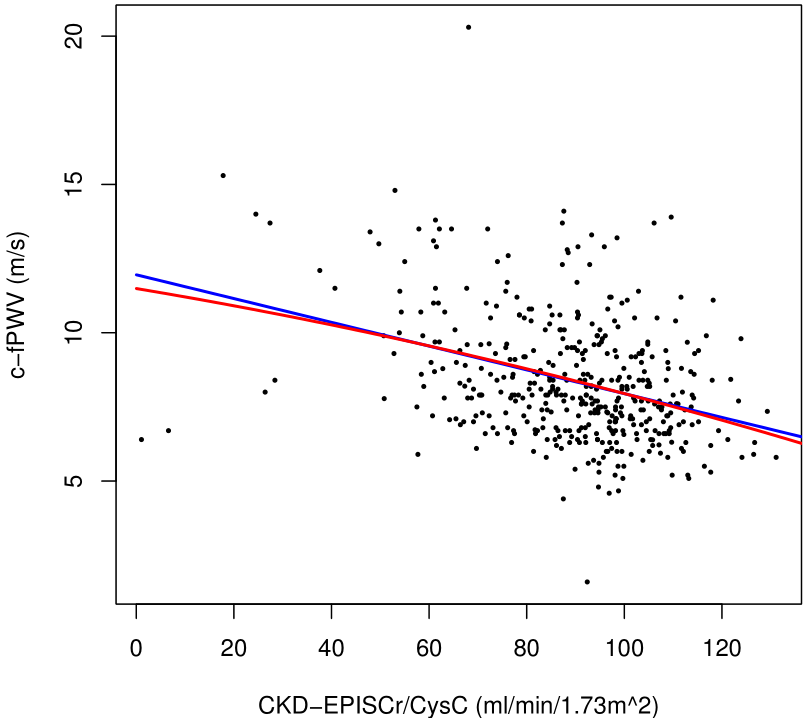


**Fig. S1**. The univariate curve fitting analyses to evaluate the correlations between CKD-EPI_CysC_, CKD-EPI_SCr/CysC_, CKD-EPI_SCr_ and MDRD and c-fPWV in health examination individuals. The red lines represent the quadratic regression model ; The blue lines represent the simple linear regression model.

**Table.S1**: The ANOVA test of the simple linear regression model and quadratic regression model

| **Parameters** | **β** | **se** | **Adjusted R^2^** | **P-value** |
| --- | --- | --- | --- | --- |
| Linear regression |  |  |  |  |
| CKD-EPI_CysC_ | -0.037979 | 0.00417 | 0.1465 | ＜0.0001 |
| Quadratic regression |  |  | 0.1479 |  |
| CKD-EPI_CysC_ | -0.06818 | 0.0235749 |  | 0.000403 |
| CKD-EPI_CysC_^2^ | 0.0001903 | 0.0001460 |  | 0.19319 |
|  |  |  |  |  |
| Linear regression |  |  |  |  |
| CKD-EPI_SCr/CysC_ | -0.040069 | 0.005091 | 0.1254 | 2.99×10^-4^ |
| Quadratic regression |  |  | 0.1239 |  |
| CKD-EPI_SCr/CysC_ | -0.02749 | 0.0245 |  | 0.262 |
| CKD-EPI_SCr/CysC_^2^ | -7.922×10^-5^ | 1.509×10^-4^ |  | 0.6 |
|  |  |  |  |  |
| Linear regression |  |  | 0.1114 |  |
| CKD-EPI_SCr_ | -0.045866 | 0.006224 |  | 9.07×10^-13^ |
| Quadratic regression |  |  | 0.1094 |  |
| CKD-EPI_SCr_ | -5.324×10^-2^ | 3.278×10^-2^ |  | 0.113 |
| CKD-EPI_SCr_^2^ | 3.899×10^-5^ | 1.949×10^-4^ |  | 0.843 |
|  |  |  |  |  |
| Linear regression |  |  | 0.04539 |  |
| MDRD | -0.027851 | 0.006041 |  | 5.23×10^-6^ |
| Quadratic regression |  |  | 0.05636 |  |
| MDRD | -0.100 | 0.0302639 |  | 0.00102 |
| MDRD^2^ | 0.0004234 | 0.0001696 |  | 0.01522 |
